# Hydrocéphalie compliquant une sarcoïdose chez un patient porteur d'une méningocèle

**DOI:** 10.11604/pamj.2015.22.284.8249

**Published:** 2015-11-23

**Authors:** Madiha Mahfoudhi, Rim Lahiani

**Affiliations:** 1 Service de Médecine Interne A, Hôpital Charles Nicolle, Tunis, Tunisie; 2 Service d'ORL. Hôpital Charles Nicolle, Tunis, Tunisie

**Keywords:** Sarcoïdose, hydrocéphalie, méningocèle, sarcoidosis, hydrocephalus, meningocele

## Image en medicine

Une méningocèle est une saillie des méninges à travers des points de faiblesse, habituellement dans la base du crâne. Elle peut être congénitale, iatrogène ou spontanée. Elle est le plus souvent asymptomatique mais peut parfois se manifester par une rhinorrhée, une otorrhée, ou des signes de méningite bactérienne récurrente. L'association à une neurosarcoïdose est exceptionnelle. Patient âgé de 37 ans, a consulté pour une rhinorrhée récidivante évoluant depuis un an. Il s'agissait d'un patient suivi depuis deux ans pour une sarcoïdose systémique dont le diagnostic a été retenu devant l'association d'une xérostomie, un lupus pernio, une pneumopathie interstitielle, une alvéolite lymphocytaire et une granulomatose sans nécrose caséeuse à la biopsie labiale et bronchique étagée; et dont le traitement s'est basée sur une corticothérapie générale avec bonne évolution clinique. L'examen physique a montré une muqueuse nasale saine. Le patient n'avait pas de syndrome méningé. L'examen ophtalmologique était normal. L'analyse du liquide nasal a confirmé qu'il s'agissait d'un liquide céphalo-rachidien. L'examen biologique et la biopsie de la muqueuse nasale étaient sans anomalies. La TDM cérébrale a révélé l'image d'une méningocèle associée à une hydrocéphalie. Le diagnostic d'une hydrocéphalie compliquant une sarcoïdose chez un patient porteur d'une méningocèle a été retenu. Le traitement s'est basé sur un traitement chirurgical de la méningocèle et un traitement médical qui a consisté en une corticothérapie associée à des boli de cyclophosphamide. L’évolution était marquée par une bonne évolution clinique et radiologique et l'absence de récidive de la rhinorrhée.

**Figure 1:**
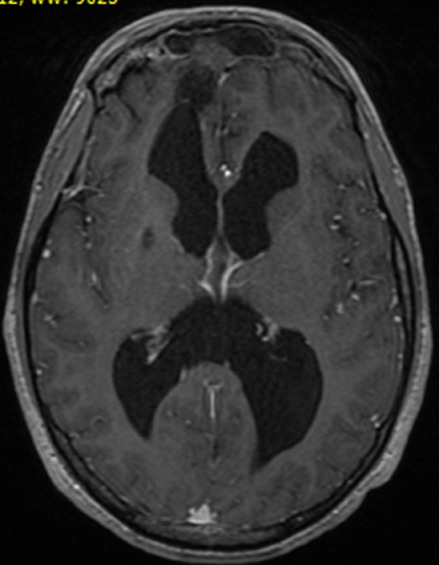
TDM cérébrale (coupe axiale): image d'une méningocèle associée à une hydrocéphalie

